# Non-response bias and hazardous alcohol use in relation to previous alcohol-related hospitalization: comparing survey responses with population data

**DOI:** 10.1186/1747-597X-8-10

**Published:** 2013-03-04

**Authors:** Kozma Ahacic, Ingemar Kåreholt, Asgeir R Helgason, Peter Allebeck

**Affiliations:** 1 Centre for Epidemiology and Community Medicine, Health Care Services, Stockholm County Council, Box 1497, Solna, 171 29, Sweden; 2 Department of Public Health Sciences, Karolinska Institutet, Tomtebodavägen 18A, Stockholm, 171 77, Sweden; 3 Karolinska Institutet and Stockholm University, Aging Research Centre (ARC), Gävlegatan 16, Stockholm, 113 30, Sweden; 4 Institute of Gerontology, School of Health Sciences, Jönköping University, Sweden, Jönköping, Sweden

**Keywords:** Non-response bias, Missing data, Attrition, Hazardous alcohol use, Abstainers, Abstinence

## Abstract

**Background:**

This study examines whether alcohol-related hospitalization predicts survey non-response, and evaluates whether this missing data result in biased estimates of the prevalence of hazardous alcohol use and abstinence.

**Methods:**

Registry data on alcohol-related hospitalizations during the preceding ten years were linked to two representative surveys. Population data corresponding to the surveys were derived from the Stockholm County registry. The alcohol-related hospitalization rates for survey responders were compared with the population data, and corresponding rates for non-responders were based on the differences between the two estimates. The proportions with hazardous alcohol use and abstinence were calculated separately for previously hospitalized and non-hospitalized responders, and non-responders were assumed to be similar to responders in this respect.

**Results:**

Persons with previous alcohol-related admissions were more likely currently to abstain from alcohol (RR=1.58, p<.001) or to have hazardous alcohol use (RR=2.06, p<.001). Alternatively, they were more than twice as likely to have become non-responders. Adjusting for this skewed non-response, i.e., the underrepresentation of hazardous users and abstainers among the hospitalized, made little difference to the estimated rates of hazardous use and abstinence in total. During the ten-year period 1.7% of the population were hospitalized.

**Conclusions:**

Few people receive alcohol-related hospital care and it remains unclear whether this group’s underrepresentation in surveys is generalizable to other groups, such as hazardous users. While people with severe alcohol problems – i.e. a history of alcohol-related hospitalizations – are less likely to respond to population surveys, this particular bias is not likely to alter prevalence estimates of hazardous use.

## Introduction

The trend in decreasing response rates in population surveys is a growing concern in epidemiological research because of the increased likelihood of non-response bias [1-4]. Low response rates threaten to compromise the generalizability of population survey data, not least because of the general underestimation of alcohol consumption when self-report measures are used in population surveys [5]. As well as affecting prevalence estimates, the resulting skewness might change associations with other variables.

Both abstainers and hazardous alcohol users seem to be overrepresented among non-responders to population surveys [6-23]. Reasons for not responding to surveys among high consumers of alcohol are likely to include frequent changes of address, failing to show up for appointments, not being at home, irregular life styles, and current drinking problems, including binge drinking and chronic heavy drinking. Some high alcohol consumers also have periods of abstinence followed by relapse. Abstinence, moreover, can be recognised as a successful outcome for people with alcohol use disorders. The issue of alcohol-related non-response bias has been dealt with by follow-up of initial non-responders, thus increasing the response rates somewhat [6,7]. Alternatively, in longitudinal surveys or clinical samples, attrition rates have been empirically evaluated [8-23]. With some exceptions [10,17,18], studies examining attrition have found that alcohol use is related to future non-response.

However, in previous studies, the issue of alcohol-related non-response has generally been examined in sub-samples of total non-response data, based on earlier or later responses. Since non-response appears to be non-random, more comprehensive evaluations may be needed so as better to understand the biases involved [24,25]. These biases can be more accurately assessed if all non-responders are considered [25].

This study examines non-response as a whole by developing population parameters corresponding to survey estimates using registry data covering all alcohol-related hospitalizations over a 10-year period. This allows us to estimate and adjust for differing hospitalization rates among non-responders and to examine possible biases in estimates of alcohol use.

The study examines whether people with alcohol-related hospitalizations are overrepresented in non-response to population surveys, and whether this results in biased estimates of the prevalence of hazardous alcohol use and abstinence.

## Material and methods

The study combines analyses of the Stockholm County registry with analyses of survey data linked to national registries in Sweden.

### The sample

The pooled study sample (n=58 506, response rate 55.3 percent) is based on two postal surveys, carried out in 2006 and 2007. The sampling frames were stratified by gender and region to allow for breakdown of the county’s different municipalities and districts. After correcting for sampling probability, persons were randomly selected to be representative of Stockholm County’s population. The 2006 survey (n=34 707, response rate 61.3 percent) was a cross-sectional study, and the 2007 survey (n=23 797, response rate 49.7 percent) was the follow-up to a longitudinal study, which was undertaken in 2002. In 2007, responders to the baseline survey in 2002 were approached again. The response rate was 62.5 percent in 2002, and the follow-up response rate was 79.6 percent in 2007.

The 2006 survey included persons born between 1922 and 1988, while the 2007 survey included those born between 1918 and 1984. In order to equate age spans at the time of follow-up, 23–84 years, the 1921–1983 birth cohorts (n=32 626) were included from the 2006 survey, and the 1922–1984 birth cohorts (n=23 369) from the 2007 survey.

Both surveys were administered by Statistics Sweden. By linking the survey responders to national registries, it was possible to include information about their alcohol-related hospitalizations. People consented to the link with the registries when responding to the surveys. Hospitalizations that occurred outside Stockholm County were disregarded.

### The population selection frames

Two population frames, corresponding to the sampling frames of the surveys above, were developed, based on Stockholm County’s registry of its inhabitants’ inpatient care. All alcohol-related care episodes in the county for the 1921–1983 cohorts in the period from 1997 to 2006, and for the 1922–1984 cohorts in the period from 1998 to 2007, were assessed. Persons who had died during the follow-up period or who had left the county were excluded (n=7333 in 1997–2006, and n=7374 in 1998–2007). To arrive at the population’s hospitalization rate, the estimated number of inpatients was divided by the corresponding population size at the end of the follow-up periods in 2006 (N=1 358 176) and in 2007 (N=1 361 698).

### Measures

#### Alcohol-related hospitalizations

The diagnoses follow the International Classification of Diseases (ICD-10) [26]. All care episodes from 1997–2006 and 1998–2007 were examined for each inpatient individually. In keeping with earlier studies, the first three diagnoses listed in all care episodes identified as alcohol-related by the National Board of Health and Welfare were evaluated [27,28]. The following alcohol-related diagnoses were considered: alcohol intoxication, corresponding to F10.0, acute intoxication due to alcohol, or T51 toxic effect of alcohol; harmful use of alcohol F10.1; alcohol dependence F10.2; alcohol-induced chronic pancreatitis K86.0; alcoholic liver disease K70; alcohol-induced pseudo-Cushing's syndrome E24.4; degeneration of nervous system due to alcohol G31.2; alcoholic polyneuropathy G62.1; alcoholic myopathy G72.1; alcoholic cardiomyopathy I42.6; alcoholic gastritis K29.2; maternal care for (suspected) damage to foetus from alcohol O35.4; foetus and new-born affected by maternal use of alcohol P04.3; foetal alcohol syndrome Q86.0; blood alcohol level Y90; alcohol intoxication Y91; alcohol rehabilitation Z50.2; alcohol abuse counselling and surveillance Z71.4; and mental and behavioural disorders due to use of alcohol (F10), i.e., withdrawal state F10.3, delirium F10.4, psychotic disorder F10.5 & F10.7, amnesic syndrome F10.6, and other mental and behavioural disorders F10.8 & F10.9.

#### Alcohol consumption

Alcohol consumption during a typical week was measured using a beverage-specific grid in the 2006 survey [29,30]. The grid consisted of four rows, with a single row comprising the first four days of the week, and separate rows for the weekend days, Friday, Saturday, and Sunday. Different columns corresponded to the different beverages: spirits, fermented wine, wine, beer, and low alcohol beer. The responders filled in the estimated volume they had consumed by day of the week and beverage. In 2007, the AUDIT-C instrument [31,32] was used instead of the grid. Both measures identified abstainers and hazardous alcohol users, and also non-hazardous users. Hazardous use is consumption in excess of 14 (men) or 9 (women) normal glasses (equivalent to 12g of pure alcohol) of alcoholic beverage per week (score 8+/6+ in AUDIT-C) [33].

#### Analysis

The estimated hospitalization rates from the surveys were compared with the population-based figures using 95% confidence intervals. Then, the hospitalization rates of non-responders were estimated. Based on the population data, the number of hospitalized among the non-responders was calculated by adding the excess numbers deriving from the difference between the surveys and population data. That is, the number of hospitalized among the responders from the surveys was subtracted from the expected number of hospitalized among the responders based on their percentage in the population data. To this number was added the expected number of hospitalized among the non-responders, based on their percentage in the population data.

Next, the numbers of abstainers, non-hazardous users, and hazardous users of alcohol were estimated for the hospitalized and non-hospitalized responders, and the difference between them was evaluated. An adjustment was made to account for possible bias due to a lower response rate among the hospitalized. These estimated alternative figures for the prevalence of abstainers, non-hazardous users, and hazardous users of alcohol took the differing hospitalization rates among the non-responders into account. It was assumed that the proportions of abstainers, non-hazardous users, and hazardous users of alcohol were the same for non-responders and responders within each stratum of hospitalization, i.e., among the hospitalized and non-hospitalized. The corrected abstainer rates for non-responders were computed by adding the numbers of non-hospitalized and hospitalized in each category of alcohol use. Lastly, the totals of responders and the non-responders were added together to arrive at the adjusted grand total for each category. In this way, it was possible to adjust the proportions for the non-responders and for the entire survey samples.

The two survey samples were first analysed separately and then pooled. The samples were stratified and weighted using SAS software (PROC SURVEYFREQ, PROC SURVEYLOGISTIC) for frequency tables and logistic regression models.

The first analysis shows both the weighted and un-weighted hospitalization rates (the latter in footnotes). This was because there were no stratification data available for the non-responders, and the later analysis had to be based on un-weighted percentages. The weighting compensated for the overrepresentation of the county’s smaller municipalities and districts in comparison with the larger ones.

In order to examine the association between previous hospitalizations and current alcohol use, logistic regression was utilised to allow for control of gender and age group. Although the relative risks became somewhat smaller due to these controls, the results were generally similar. Accordingly, rather than presenting odds ratios, only the relative risks without control are presented.

## Results

Table 1 shows that, of the responders in the 2006 survey, 1.1 percent had been hospitalized during the preceding ten years. When compared with the population parameters, both surveys indicated significantly lower hospitalization rates. The resultant estimates for the non-responders are also shown.

**Table 1:** **Percentages of people with alcohol**-**related hospitalization in the surveys**, **and in the corresponding population data**, **and the resulting estimates of non**-**responders in the surveys**

**Material**	**Percent**	**95% CI**^**1**^	***Hospitalized n***	***Total N***
2006 cross-sectional survey	1.13^2^	0.99, 1.22	*362*	*32 626*
2006 population data	1.64		*22 298*	*1 358 176*
2006 non-responders	2.48^3^		*511*	*20 597*
2002-2007 longitudinal survey	0.92^4^	0.78, 1.02	*210*	*23 369*
2007 population data	1.68		*22 841*	*1 361 698*
2007 non-responders	2.45^3^		*580*	*23 651*
Both surveys	1.04^5^	0.95, 1.14	*572*	*55 995*
Population data	1.66		*22570*	*1 359 937*
Non-responders	2.47^3^		*1119*	*45 262*

^1^Confidence intervals are given for the survey responders, since it is their estimates that are being compared with the corresponding population parameters.

^2^ Since the surveys were stratified by gender, municipality and district, this estimate was weighted for sampling probability. The corresponding un-weighted estimate was 1.11 percent.

^3^ The estimate of the non-responders was based on un-weighted estimates of the responders. It was obtained by assuming that survey estimates correspond to population data.

^4^ The un-weighted estimate was 0.90 percent.

^5^ The un-weighted estimate was 1.02 percent.

The relative risks suggest that the persons who had been hospitalized were 2.2 times more likely to have become non-responders in the 2006 study, and 2.7 times more likely in the 2002–2007 study. Taken together, persons with previous alcohol-related hospitalization (either once or several times) were, on average, 2.4 times more likely to have become survey non-responders than others.

Table 2 shows the association between previous hospitalization and current alcohol use. Previously hospitalized responders were more likely to abstain from alcohol (relative risk, RR=1.58). They were also more likely to have engaged in hazardous alcohol use (RR=2.06), and less likely (RR=0.68) to have non-hazardous use. In further analyses (not shown here), the same pattern of results was obtained after controlling for gender and age group.

**Table 2:** **Percentages of abstainers**, **and of non**-**hazardous and hazardous alcohol users among the survey responders**, **with corresponding estimates of non**-**responders**, **adjusted for the differing alcohol**-**related hospitalization rates**

	**Responders**				**Non-responders**^**1**^	**Adjusted estimate**^**1**^
**Material**	**non-hospitalized %**	**hospitalized**		**all %**	**all %**	**all %**
		**%**	**RR**			
***2006 cross***-***sectional sample***						
Abstainers	11.9	18.3	1.54^**^	12.0	12.1	12.0
Non-hazardous users	66.8	48.1	0.72^***^	66.6	66.3	66.5
Hazardous users	21.3	33.6	1.58^***^	21.4	21.6	21.5
	100.0	100.0		100.0	100.0	100.0
Internal missing values	0.9	1.8	2.01	0.9		
Not missing	99.1	98.2	0.99	99.1		
	100.0	100.0		100.0		
***2002***-***2007 longitudinal sample***						
Abstainers	9.2	14.8	1.61^*^	9.2	9.3	9.3
Non-hazardous users	82.3	53.2	0.65^***^	82.1	81.6	81.8
Hazardous users	8.5	32.0	3.76^***^	8.7	9.1	8.9
	100.0	100.0		100.0	100.0	100.0
Internal missing values	3.1	9.0	2.88^***^	3.2		
Not missing	96.9	91.0	0.94^***^	96.8		
	100.0	100.0		100.0		
***Both samples***						
Abstainers	10.8	17.1	1.58^***^	10.9	10.9	11.0
Non-hazardous users	73.2	49.9	0.68^***^	73.0	72.6	72.8
Hazardous users	16.0	33.0	2.06^***^	16.2	16.4	16.3
	100.0	100.0		100.0	100.0	100.0
Internal missing values	1.8	4.6	2.48^***^	1.9		
Not missing	98.2	95.4	0.97^***^	98.1		
	100.0	100.0		100.0		

^*^p < 0.05 ^**^ p < 0.01 ^***^ p < 0.001 significance levels are given for the hospitalized in comparison with the non-hospitalized, using Wald chi-square tests for logistic regression models.

^1^ The estimated rates of abstainers, and non-hazardous and hazardous alcohol users among the non-responders, were assumed to be the same as among the responders within each stratum of hospitalization, i.e., among persons with and without previous alcohol-related hospitalization. The non-responders’ rates were adjusted only for their greater likelihood of previous hospitalization (see Table 1). This adjustment was based on un-weighted numbers. Otherwise, weighted estimates which compensated for the stratification by gender, municipality, and city district were used in the table.

In 2007, those previously hospitalized were also more likely to avoid answering the survey’s alcohol-related items.

Table 2 also suggests that adjusting for a differing pattern of alcohol use among non-responders had only a marginal effect on the estimated percentages of alcohol use.

## Discussion

Persons who previously had been hospitalized for alcohol-related reasons were more likely than others to become non-responders in population health surveys, but adjusting for this particular source of non-response bias made little difference to the estimated prevalence of hazardous use or of abstinence.

The results were in line with previous studies [6-16], demonstrating higher risks (odds ratios up to 2.0) of becoming non-responders among excessive alcohol users. These studies have used alcohol consumption rather than hospitalization as a predictor of non-response, and had varying definitions of consumption levels and/or excessive drinking. In general, they have examined different sub-populations of all non-responders. By contrast, the current study analysed the entire samples, corresponding to a 100% response rate. It therefore also included the persons least likely to be reached by representative surveys. Examining non-random samples of non-responders can be misleading, which possibly explains the sometimes contradictory findings of previous studies. Our study indicates somewhat larger ORs than those reported in previous studies, which might suggest that non-response related to alcohol use is underestimated in designs based on earlier or later responses. But it might also reflect people with severe alcohol problems being more likely to become non-responders than people with less of a problem.

The current study also considered the hypothesis that previous hospitalization predicts current alcohol use. Only the opposite relationship has been shown previously [34]. Further analysis and discussion of this particular result may be found in an accompanying article [35]. In this context, alcohol-related hospitalization can reasonably be seen as a marker of deviant alcohol habits.

During the decades investigated, fewer than one in fifty adults received alcohol-related hospital care. Thus, only a small proportion of the population suffered this particular adverse consequence of their drinking behaviour. A history of alcohol-related morbidity, indicated here by hospitalization, suggests a life style of abuse and deviance. To our knowledge, the extent to which such a population is reached by general representative population surveys has not been examined previously. This is also the first study to present hospitalization rates over a period of time as long as ten years.

Although the bias caused by a small sub-population of non-responders may be rather modest, the true alcohol-related reporting bias may be larger than the adjustment for the non-response hospitalization rate in our study suggests. Only a small proportion of the population with alcohol problems receive hospital care for alcohol-related causes; most persons with alcohol-use disorders go untreated or undetected by the health care system [36]. Also, few studies have examined the relationship between alcohol-related hospitalization and alcohol use [34]. Thus, it is not clear whether people who receive alcohol-related hospital care resemble the larger population of people with alcohol problems with respect to reporting behaviour. It is, therefore, also unclear whether our results on those who have been hospitalized generalize to all people with alcohol-use disorders or possibly to an even larger population, such as hazardous users. Future studies may wish to examine hospitalization, alongside abstinence and hazardous use, as predictors of attrition. If hazardous users are also twice as likely to become non-responders than others, the prevalence of hazardous use may be seriously underestimated.

### Limitations

Non-response is likely to be related to factors other than hospitalization, such as gender, age, social class, etc., some of which are also likely to covary with alcohol use. A limitation to the study was that it was restricted solely to examining the bias from a different hospitalization rate among non-responders. Yet, there are other biases that probably affect estimated prevalence rates.

Self-rated alcohol consumption figures capture about half the population’s actual consumption [5]. Like other studies of self-rated alcohol consumption, this study rests on the assumption that underrating in self-reports is proportionately distributed but otherwise random. But it remains unclear whether this is true. Underreporting may differ by sub-group; for example, heavy consumers may underreport to a greater degree.

The assumption of similar alcohol use among non-responders and responders for the different strata of hospitalization is questionable. But it was mainly because of the small proportion of the population hospitalized (just 1.7%) that the effects on estimated prevalence of hazardous use and of abstinence were insignificant. Even if hazardous alcohol use was more widespread among non-responders than among responders, the effects on prevalence would still have been small.

In this longitudinal study, consent to link response data with registry data was not sought from responders until follow-up in 2007, which meant that the hospitalization rate for non-responders between 2002 and 2007 was not available. It was, therefore, not possible to differentiate between non-response and attrition in the analyses. Although attrition is likely to contribute to selection bias, researchers usually have some information to go on, e.g., from baseline measures, and are therefore in a position to analyse it. Further, attrition bias can be mitigated because there are usually variables available that can be used to treat the missing data.

## Conclusions

This study found that people with alcohol-related morbidity, i.e., the previously hospitalized, were twice as likely to become non-responders as people in the general population. It demonstrates that those who suffer the adverse consequences of alcohol abuse are reached in representative population surveys, albeit to a lesser extent than others. The study also illustrates that the effect of non-response bias associated with previous alcohol-related hospitalization is fairly small, or even trivial, on the estimated prevalence rates of hazardous alcohol use and abstinence.

## Competing interest

The authors are not aware of any conflicts of interest.

## Authors’ contributions

KA conceived the study, performed the statistical analysis, and wrote the draft of the manuscript. PA is responsible for the part about alcohol in the Stockholm County Public Health Surveys, and his concern about data quality was the study’s starting point. IK was the statistical adviser, and ARH is an expert in the area of treatment effects. All the four authors revised the draft critically for important intellectual content, and they have all read and approved the final manuscript.
